# Constructing prediction models from expression profiles for large scale lncRNA–miRNA interaction profiling

**DOI:** 10.1093/bioinformatics/btx672

**Published:** 2017-10-23

**Authors:** Yu-An Huang, Keith C C Chan, Zhu-Hong You

**Affiliations:** 1 Department of Computing, Hong Kong Polytechnic University, Hong Kong, China; 2 Xinjiang Technical Institute of Physics and Chemistry, Chinese Academy of Science, Ürümqi, China

## Abstract

**Motivation:**

The interaction of miRNA and lncRNA is known to be important for gene regulations. However, not many computational approaches have been developed to analyze known interactions and predict the unknown ones. Given that there are now more evidences that suggest that lncRNA–miRNA interactions are closely related to their relative expression levels in the form of a titration mechanism, we analyzed the patterns in large-scale expression profiles of known lncRNA–miRNA interactions. From these uncovered patterns, we noticed that lncRNAs tend to interact collaboratively with miRNAs of similar expression profiles, and vice versa.

**Results:**

By representing known interaction between lncRNA and miRNA as a bipartite graph, we propose here a technique, called EPLMI, to construct a prediction model from such a graph. EPLMI performs its tasks based on the assumption that lncRNAs that are highly similar to each other tend to have similar interaction or non-interaction patterns with miRNAs and vice versa. The effectiveness of the prediction model so constructed has been evaluated using the latest dataset of lncRNA–miRNA interactions. The results show that the prediction model can achieve AUCs of 0.8522 and 0.8447 ± 0.0017 based on leave-one-out cross validation and 5-fold cross validation. Using this model, we show that lncRNA–miRNA interactions can be reliably predicted. We also show that we can use it to select the most likely lncRNA targets that specific miRNAs would interact with. We believe that the prediction models discovered by EPLMI can yield great insights for further research on ceRNA regulation network. To the best of our knowledge, EPLMI is the first technique that is developed for large-scale lncRNA–miRNA interaction profiling.

**Availability and implementation:**

Matlab codes and dataset are available at https://github.com/yahuang1991polyu/EPLMI/.

**Supplementary information:**

Supplementary data are available at *Bioinformatics* online.

## 1 Introduction

The discovery of the essential role of non-coding RNAs (ncRNAs) in the regulation of gene expression leads many to believe that the transcriptional landscape of many organisms is far more complex than previously thought (Salmena *et al.*, 2011). ncRNAs, in the vast majority of transcripts expressed in mammals, have lengths ranging from 22 nucleotides to hundreds of kb. The *long ncRNA* (lncRNA) among the ncRNAs is a loosely classified group of RNA transcripts longer than 200 bases with no apparent protein-coding function and they can be found in every branch of life (Volders *et al.*, 2013). There has recently been increasing evidence that lncRNAs can be involved in various cellular processes, such as cell differentiation, cell growth and death etc. They seem to be able to exert influences over chromatin modification, transcriptional complex targeting, mRNA splicing and protein translation. The past few years have witnessed a surge of interest in the development of computational tools for the identification and annotation of ncRNA (Liu *et al.*, 2015a,b, 2016a,b). However, even though more than 58 000 human lncRNA genes have been identified, apart from the few lncRNAs, like XIST and HOTAIR, that are well-studied, the role that most lncRNAs can play in different cellular processes remain largely unknown due to the complex and dynamic molecular mechanisms (Quinn and Chang, 2016).

LncRNAs have been found to be able to regulate patterns of expressed proteins via a specific mechanism composed of different kinds of biological interactions such as the interactions between lncRNA and protein, lncRNA and mRNA, and lncRNA and ncRNA (Li *et al.*, 2013). As a result, the construction of maps of putative biological interaction network mediated by lncRNAs could be necessary for the understanding of potential biological functions and mechanisms of lncRNAs. As a main kind of competing endogenous RNAs (ceRNAs), lncRNAs can function as miRNA sponges, leading to lower regulatory effect of miRNA on mRNAs, and therefore miRNAs play significant roles in the molecular mechanisms of lncRNAs (Salmena *et al.*, 2011). Previous work on function annotation of lncRNAs are mainly based on expression correlation between lncRNAs and protein-coding genes across different tissues (Cabili *et al.*, 2011; Derrien *et al.*, 2012). Few functional annotations were conducted based on the ceRNA network. Given the knowledge accumulated over miRNA function for the past decade, if the interaction between lncRNA and miRNA can be better understood or even predicted, we can gain great insights into the complex functions of lncRNA.

Recently, there are more and more evidence to show that both miRNA and lncRNA are implicated in the pathological processes involved in diverse human diseases. And as a result, there has been much effort to investigate into the impacts that miRNA can have on lncRNA functions and vice versa (Quinn and Chang, 2016; Yoon *et al.*, 2014). For example, lncRNA–miRNA regulatory networks in prostate cancer, gastric cancer and vascular diseases have been constructed (Ballantyne *et al.*, 2016; Du *et al.*, 2016; Xia *et al.*, 2014) have been studied. Such detailed understanding of the effects of lncRNA–miRNA interactions can have in pathophysiology could pave the way for new biomarker discovery and therapeutic approaches. Unfortunately, however, the interaction between lncRNA–miRNA as identified by biological experiments is still too limited for such understanding to make very wide impacts.

To expedite the process of identifying such miRNA–target interactions, it is common practice to perform *in silico* prediction to refine the candidate list for further validation experiments (Poliseno and Pandolfi, 2015). Existing computational algorithms developed for predicting such miRNA–target predictions are designed with several common rules that address the four aspects of conservation, seed match, free energy and site accessibility (Quinn and Chang, 2016). However, many miRNA–target prediction tools are developed originally for mRNA targets, and as a result, predictions are made based on the nature and statistical rules of mRNA–miRNA interactions and may contradict with that of lncRNA–miRNA interactions (Li *et al.*, 2015a,b). For example, some existing prediction methods for miRNA–target interactions perform conservation analysis focusing on the regions in the 3′ and the 5′ UTR of mRNA based on the observation that the miRNA seed region of mRNA usually has higher conservation than the non-seed regions. However, lncRNA is reported to show significantly lower sequence conservation and evolve faster than mRNAs (Quinn and Chang, 2016).

In addition to this, it is also noted that as the strategy of seed match is based on the statistical rules originally obtained for miRNA–mRNA interactions, they would be unsuitable for lncRNA–miRNA interaction prediction.

Besides, a few models proposed for prediction of lncRNA–RNA interaction perform their tasks by simply computing the free energy of the potential-binding sites (Quinn and Chang, 2016). For example, LncTar computes the free energy which is to measure the stability of complementarity between lncRNA and target RNA (Li *et al.*, 2015a,b). However, although this kind of sequence-based prediction algorithm has a wide application range, they are plagued by very high false positive rates (FPRs) (Poliseno and Pandolfi, 2015). Other than this, some inherent characteristics are found to differentiate lncRNAs from mRNAs. For example, comparing with mRNAs, lncRNAs are generally found to be shorter with fewer exons. They are also more lowly expressed, more enriched in the nucleus, and show higher tissue-specificity and reduced stability (Quinn and Chang, 2016). Most existing miRNA target prediction tools fail to incorporate recent advancements in the understanding of lncRNA–miRNA interaction and may therefore not effective enough for the prediction of lnRNA/miRNA targets for a specific miRNA/lncRNA.

Recent theoretical and experimental research have shed light on the modeling of the crosstalk between different kinds of ceRNAs, including lncRNA and miRNA within the cell (Cesana and Daley, 2013). It appears that, apart from other well-known factors such as sub-cellular localization and miRNA response element (MRE) accessibility associated with secondary structures or RNA-binding protein, the expression levels of individual lncRNA and miRNA has come to be the key to decipher the rules of ceRNA networks (Ala *et al.*, 2013).

Previous work on protein–protein interaction predictions (Buchler and Louis, 2008), small RNA regulation (Levine and Hwa, 2008) and miRNA–target threshold effects (Mukherji *et al.*, 2011) reveal that, as the two major components of ceRNA network, lncRNAs and miRNAs interact with each other according to a titration mechanism which orchestrates their interaction by establishing a threshold level of effect. The basic postulate of this titration mechanism is that optimal lncRNA–miRNA cross-regulation occurs at a near-equimolar equilibrium because lncRNA would be inactive in the presence of limited number of available miRNA and, conversely, be fully repressed when miRNA molecules are much more abundant (Ala *et al.*, 2013). In other words, RNA dosage is critical for cross-regulation and the baseline expression levels of miRNA and lncRNA can offer important insights into their direct and indirect interaction patterns according to the overall network equilibrium.

Based on such considerations, Ala et al. proposed a kinetic mathematical model to predict ceRNA interactions mediated by phosphatase and tensin homolog. This kinetic model makes use of transcription and degradation rates for miRNA/ceRNAs association/dissociation and the degradation rates for miRNA/ceRNA complexes as the model’s key parameters (Ala *et al.*, 2013). However, all these parameters are hard to be defined for most lncRNAs and miRNAs and as a result, the kinetic model cannot be widely used for predicting lncRNA–miRNA interactions. The result of Ala’s work demonstrates that ceRNA crosstalk has a close relationship with the expression levels of relative miRNAs, and the specificity of ceRNA interactions may depends on the expression profiles of miRNA.

There are increasing evidences that certain lncRNAs are presumably co-regulated in expression networks, suggesting that multiple lncRNAs may regulate biological processes through interacting with specific miRNA clusters in a synergistic manner (Li *et al.*, 2015a,b; Yang *et al.*, 2016). It may reasonably be assumed that there is lncRNAs interacting with same miRNAs are expressed similarly across different tissues and cell lines.

Therefore, we investigated into the expression patterns of a large number of lncRNA–miRNA interactions identified by high-throughput experiments, and have discovered that the miRNAs that have been identified to interact with specific lncRNA tend to share more similar expression pattern than those are not known to be interactive. Conversely, the expression profiles of lncRNAs that have been identified to interact with the same miRNA also tend to be more similar than those of the others.

Motivated by this discovery and the limited knowledge known about MRE-binding rules, we propose here a computational model to predict large-scale lncRNA–miRNA interaction network as a whole. To the best of our knowledge, this is the first of its kind.

Without using the sequence data of lncRNAs and miRNAs, we develop a two-way diffusion model called EPLMI to predict new lncRNA–miRNA interactions and compute the putative interaction strength of known lncRNA–miRNA interactions based on known lncRNA–miRNA interaction network. The basic assumption behind the development of the model is that lncRNAs of similar expression profiles tend to interact with a cluster of miRNAs having similar expression profiles, and vice versa.

To evaluate the performance of the proposed model, we implemented 5-fold cross validation and leave-one-out cross validation (LOOCV) to predict lncRNA–miRNA interactions using the dataset collected from the most up-to-date version of the lncRNASNP database. We performed, in addition, a number of experiments for performance comparisons. In these experiments, we investigated into gene-based miRNA similarity, putative lncRNA functional similarity, sequence similarity and also compared the proposed method with some classical algorithms. By considering the expression profile-based similarities of lncRNAs and miRNAs, EPMDA yielded the best performance with AUCs of 0.8522 and 0.8447 ± 0.0017 based on LOOCV and 5-fold cross validation, respectively.

The experimental results suggest that Expression Profile-based prediction model for LncRNA-MiRNA Interactions (EPLMI) is feasible and effective for predicting large-scale lncRNA–miRNA interactions and for measuring the competitiveness of lncRNAs identified to interact with specific miRNAs.

## 2 Materials

For the purpose of our investigation, we obtained the February 2017 version of the lncRNASNP database which is made available for downloading at http://bioinfo.life.hust.edu.cn/lncRNASNP. The database contains information about known lncRNA–miRNA interactions confirmed by laboratory studies (Gong *et al.*, 2015). They were collected from 108 CLIP-Seq datasets and there are 8091 records in total. After removing the duplicated entries, we obtained 5348 of them for our experiments. These records represent lncRNA–miRNA interactions, involving 780 different types of lncRNAs and 275 different types of miRNAs, respectively.

In addition, for the purpose computing the similarities among miRNAs, we have collected three kinds of information from various databases. The first of such information is related to the interaction between miRNAs and different target genes and is obtained from miRTarBase (release 6.1, http://miRTarBase.mbc.nctu.edu.tw) (Chou *et al.*, 2015; Hsu *et al.*, 2010). After matching the ids of the 275 types of miRNAs, we managed to obtain information on 272 of them.

The second type of information is obtained from the expression profile data of miRNAs. The data were downloaded from the microRNA.org database (http://www.microrna.org/microrna/home.do) where the expression profiles of 230 miRNAs were found (Betel *et al.*, 2008). Each record of miRNA expression profile has 172 dimensions representing the expression levels of a single type of miRNAs in 172 different human tissues and cell lines.

The third type of information is obtained from the sequence data of mature miRNAs. The data were obtained from the miRBase database (http://www.mirbase.org/index.shtml) (Kozomara and Griffiths-Jones, 2014).

To compute the similarity among lncRNAs, we downloaded the putative functional annotations of lncRNAs from the NONCODE database (http://www.noncode.org/) (Bu *et al.*, 2011). After converting the names of lncRNA into the NONCODE IDs, we successfully obtained expression profile data for 450 of the lncRNAs and the functional annotations of 264 of the lncRNAs.

The collected expression profiles of lncRNA have 22 properties describing the expression level of each type of lncRNAs in 16 different human tissues and 8 cell lines. The functional annotations for lncRNA genes we obtained describe the 10 most probable biological functions as predicted by lnc-GFP method based on a coding–non-coding co-expression network.

Finally, for performance assessment, we have also downloaded the sequence data of lncRNAs from LNCipedia database (https://lncipedia.org/) (Volders *et al.*, 2013).

## 3 Methods

### 3.1 Construction of diverse lncRNA/miRNA similarity matrixes

Based on the assumption that lncRNA/miRNA tends to interact with a cluster of miRNAs/lncRNAs which share similar features and regulation patterns, we have investigated into three different types of lncRNA/miRNA similarity by incorporating diverse information resources. The similarity matrix we computed for the first type is based on the data of expression profiles. Specifically, we use Pearson correlation coefficient (PCC) for similarity measurement. Given two expression profiles of two RNAs (say *e*_a_ and *e*_b_), the correlation coefficient score is computed as follow:

(1) $$\text{ES}(a,b)=\frac{\sum_{i=1}^{N}\left(e_{ai}-\bar{e_{a}})(e_{bi}-\bar{e_{b}}\right)}{\sqrt{\sum_{i=1}^{N}{\left(e_{ai}-\bar{e_{a}}\right)}^{2}\sum_{i=1}^{N}{\left(e_{bi}-\bar{e_{b}}\right)}^{2}}}$$

where *N* denotes the number of properties of the expression profiles and is 172 for miRNAs and 22 for lncRNAs. A pair of RNAs with a higher correlation score is considered to be more similarly expressed in general.

The second kind of RNA similarity we used is based on putative biological functions. Based on the assumption that miRNAs targeting more of the same genes tend to be involved in similar biological functions, the data of miRNA–target gene interactions are used to measure how functionally similar each miRNA–miRNA pair is. Given two sets of target genes respectively associated with miRNA *m*_a_ and miRNA *m*_b_ (say *G*_a_ and *G*_b_), we compute a functional similarity measure as follow:

(2) $$\text{FS}(m_{a},m_{b})=\frac{\text{card}(G_{a}\cap G_{b})}{\sqrt{\text{card}(G_{a})}\cdot \sqrt{\text{card}(G_{b})}}$$

Similarly, given two sets of putative functional annotations of two lncRNAs(say *F*_a_ and *F*_b_), their functional similarity can be computed as follow:

(3) $$\text{FS}(l_{a},l_{b})=\frac{\text{card}(F_{a}\cap F_{b})}{\sqrt{\text{card}(F_{a})}\cdot \sqrt{\text{card}(F_{b})}}$$

To compute the sequence similarity of lncRNAs and miRNAs, we implemented the Needleman-Wunsch pairwise sequence alignment by using the package of *pairwise2* in *Biopython* (Cock *et al.*, 2009). Specifically, we set the identification score, gap-open penalty and gap-open extending penalty as 2, −0.5 and <0.1, respectively (Cock *et al.*, 2009).

### 3.2 EPLMI: a graph-based method based on two-way diffusion

In recent years, the data of known lncRNA–miRNA interactions are being accumulated along with the development of high-throughput biotechnology, such as CLIP-seq. However, known lncRNA–miRNA interaction network is far from being completed due to the dynamic nature of the regulatory mechanism of miRNAs. Here, we propose a graph-based prediction method to infer the most potential lncRNA–miRNA interactions based on known lncRNA–miRNA interaction network, lncRNA–lncRNA similarity and miRNA–miRNA similarity. Specifically, the interaction data are represented by a bipartite graph between lncRNA and miRNA nodes, with identified interactions represented by links. The absence of a link would be considered as a potential interaction between an lncRNA and a miRNA that have not yet been experimentally confirmed. The task of lncRNA–miRNA interaction prediction can thus be mapped to predicting links in the bipartite graph of known lncRNA–miRNA interactions, labeled with prediction scores.

**Fig. 1.**
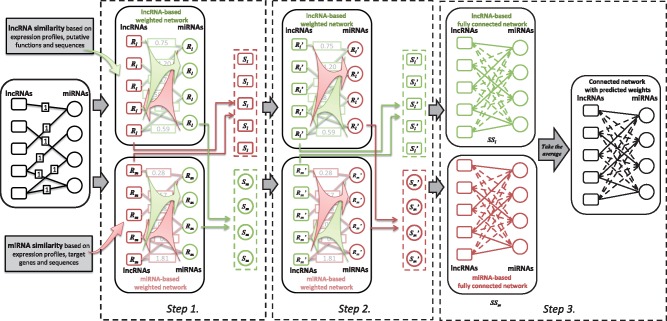
The flowchart of prediction process of EPLMI

In the prediction process of EPLMI, message flow forward and backward from one side of bipartite graph to another based on a two-way diffusion method (see Fig. 1). Specifically, EPLMI performs its tasks in three main steps. In the first step, two kinds of weighted lncRNA–miRNA interaction networks are generated in order to introduce lncRNA/miRNA similarity into the known lncRNA–miRNA interaction network. Given the corresponding adjacency matrix ***A***∈ℝ^*nl*^^×^^*nm*^ of the known lncRNA–miRNA interaction network, the lncRNA similarity matrix ***LS***∈ℝ^*nl*^^×^^*nl*^ and the miRNA similarity matrix ***MS***∈ℝ^*nm*^^×^^*nm*^, two adjacency matrixes for two weighted networks is computed as follow:

(4) $$A^{l}=\text{LS}\cdot A$$

(5) $$A^{m}=A\cdot \text{MS}$$

where *nl* and *nm* respectively denote the numbers of lncRNAs and miRNAs in the dataset. The entity ***A^l^(i, j)*** in ***A^l^*** denotes the total sum of the similarity between the *i*th lncRNA and those lncRNAs interacting with the *j*-th miRNA. Similarly, ***A^m^(i, j)*** in ***A^m^*** denotes the total sum of similarity between the *j*th miRNA with those miRNAs interacting with the *i*th lncRNA. Based on the weighted lncRNA–miRNA interaction networks, the resource vectors for both lncRNA and miRNA are further computed as follow:

(6) $$R_{{\text{lncRNA}}_{a}}=\sum_{m=1}^{nm}\frac{A_{a,m}^{w}\cdot A_{*,m}}{\sum_{i=1}^{nl}A_{i,m}^{w}}$$

(7) $$R_{{\text{miRNA}}_{b}}=\sum_{l=1}^{nl}\frac{A_{l,b}^{w}\cdot A_{l,*}}{\sum_{i=1}^{nm}A_{l,i}^{w}}$$

Here, ***A^w^*** denotes the weighted adjacency matrixes which could be either ***A^l^*** or ***A^m^***. We further encode the correlation between one type of miRNA/lncRNA and all types of lncRNA/miRNA as a resource vector. Specifically, the resource vectors for lncRNAs, i.e. ***R*_lncRNA_**, are actually *nm*-dimension row vectors and miRNA resource vectors, i.e. ***R*_miRNA_**, are *nl*-dimension column vectors, which describe the correlation scores during forward propagation. We further set the resource vectors for Step 2, i.e. ***S*_lncRNA_** and ***S*_miRNA_**, as the average of those computed based on two weighted networks. In the second step, the message flow backward to the side it starts in Step 1. To obtain the correlation scores during backward propagation, the resource vectors for lncRNA and miRNA are computed based on ***S*_lncRNA_** and ***S*_miRNA_** as follows:

(8) $$R\prime_{l{\text{ncRNA}}_{a}}=\sum_{m=1}^{nm}\frac{A_{a,m}^{w}\cdot S_{{\text{miRNA}}_{m}}}{\sum_{i=1}^{nl}A_{i,m}^{w}}$$

(9) $$R\prime_{{\text{miRNA}}_{b}}=\sum_{l=1}^{nl}\frac{A_{l,b}^{w}\cdot S_{{\text{lncRNA}}_{l}}}{\sum_{i=1}^{nm}A_{l,i}^{w}}$$

Two types of resource vectors are further combined as the resource vectors for the third step, i.e. ***S’*_lncRNA_** and ***S’*_miRNA_**, by simply taking the average. In the third step, the resource vectors of lncRNA and miRNA are respectively concatenated as two *nl* × *nm* matrixes, ***SS*_lncRNA_** and ***SS*_miRNA_**, which are correspond to two fully connect networks (see Step 3 in Fig. 1):

(10) $$SS\text{lncRNA}={\left[S^{\prime }{\text{lncRNA}}_{1}^{T},S^{\prime }{\text{lncRNA}}_{2}^{T},...,S^{\prime }{\text{lncRNA}}_{nl}^{T}\right]}^{T}$$

(11) $$SS\text{miRNA}=[S\prime {\text{miRNA}}_{1},S\prime {\text{miRNA}}_{2},\ldots ,S\prime {\text{miRNA}}_{nm}]$$

As a result, the final predict network could be computed with the average of ***SS*_lncRNA_** and ***SS*_miRNA_** as its adjacency matrix **SS**:

(12) $$\text{SS}=\frac{\text{SSlncRNA}+\text{SSmiRNA}}{2}$$

## 4 Results

### 4.1 Comparison of expression profiles between identified and unidentified lncRNA–miRNA interactions

For the purpose of assessing the effectiveness of EPLMI, we have investigated into the differences in the correlation of the expression profiles between identified and unidentified lncRNA–miRNA interactions. Based on known lncRNA–miRNA interaction network, we compared the differences in the expression profiles of two groups of miRNA/lncRNA pairs: (i) connected and (ii) unconnected miRNAs/lncRNAs for each single lncRNA/miRNA.

For each miRNA node that has more than two links, we divide the lncRNAs into two groups which we refer to as the identified miRNA group and the unidentified miRNA group, according to whether it is identified to interact with the miRNA or not. For each of the two groups, we computed the average PCCs of the expression profiles in the group.

**Fig. 2.**
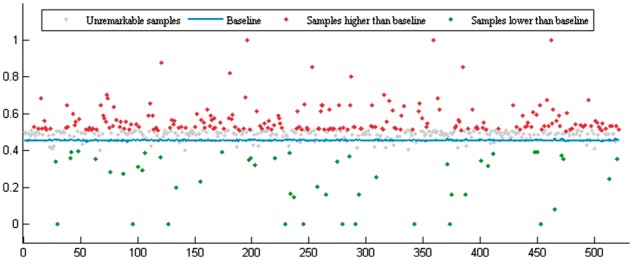
Correlation of miRNA clusters interacting with single lncRNAs

For the purpose of comparison, we used the average PCC of the unidentified group as the baseline score for each lncRNA. We noted as a result that ∼83.50% of the lncRNAs (435/521) tend to cooperate with a cluster of miRNAs sharing more similar expression profiles than the baseline (see Fig. 2). For the 521 types of lncRNAs, the average PCC value of their identified miRNA groups reaches 0.4947, which is significantly higher than the average baseline value of 0.4551. In addition, if we are to highlight the samples having significantly higher or lower PCC than the baseline by using a difference threshold of 0.5 times standard deviation of PCC of identified miRNA groups (i.e. 0.058), we can see that 80.16% (202/252) of marked samples higher than the baseline (see Fig. 2). 

Considering that a number of lncRNAs expression profiles are unavailable and that the miRNAs in our dataset are found to have interaction with an average of approximately 19 types of lncRNA, we therefore focus only on those 206 well-studied miRNAs that have more than five links in order to obtain more reliable conclusions.

**Fig. 3.**
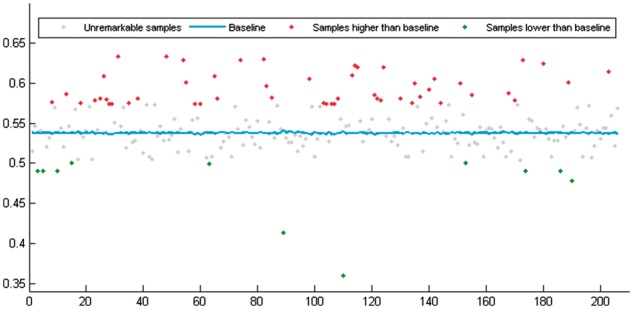
Correlation of lncRNA clusters interacting with single miRNAs

By similar analysis with both the identified lncRNA group and the unidentified lncRNA group for each single miRNA, we found that ∼59.22% (122/206) of the miRNAs tend to interact with a cluster of lncRNAs that have more strongly correlated expression profiles than the baseline (see Fig. 3). 

The average PCC of the identified lncRNA groups of 206 samples is 0.5476, which is higher than that of the baseline of 0.5378. The outstanding samples which have a different standard deviation of PCC of the identified lncRNA groups (i.e. 0.0368) from the baseline can be highlighted and shown in Figure 3. Approximately 82.26% (51/62) highlighted samples were found to be consistent with our assumption that the target lncRNAs of a specific miRNA tend to have similar expression level patterns among different tissues and cell lines.

These results confirm the influence of expression profiles of both lncRNAs and miRNAs on their pairwise interactions. Specifically, lncRNA molecules tend to interact with a cluster of miRNAs that have similar expression profiles. In addition, we also found that most of the miRNAs are targeted by lncRNA clusters which have similar expression profiles when setting a difference threshold. However, it is note-worthy that, without setting a different threshold, only <60% miRNA samples are consistent with the conclusion we made. The reason of this relatively small percentage may lie in the recent finding that lncRNA displays high natural expression variation among different individuals and therefore the expression profile data we obtained may be unrepresentative (Kornienko *et al.*, 2016). Besides, due the general lncRNA feature of high tissue-specific expression, 22 dimensions of the explored lncRNA expression profile data may not be enough for comprehensively describing the expression patterns of a single lncRNA.

To further evaluate the correlation patterns of lncRNA and miRNA with respect to other kinds of lncRNA/miRNA similarity patterns, an analogous analysis was also carried out with the functional and sequential similarities. We regard those samples obtaining higher correlation scores than the baseline as positive samples that are consistent with the basic assumption of EPLMI. As a result, 33.33% miRNA samples and 56.13% lncRNA samples are positive in the functional similarity-based experiment while 51.78% miRNA samples and 89.36% lncRNA samples are positive in the sequence similarity-based experiment.

### 4.2 Performance evaluation for EPLMI

To evaluate the accuracy of the prediction models built by EPLMI, we used a real dataset involving confirmed lncRNA–miRNA interactions and tested accuracy using the two methods of *LOOCV* and *5-fold cross validation*.

Specifically, according to LOOCV, each known lncRNA–miRNA interaction was left out, in turn, for testing and the rest of the known lncRNA-miRNA interactions were used as training samples to construct a prediction model. To avoid the denominators in formulas (6–9) becoming zeros, we replace all zeros in ***A^w^*** with a tiny value of *10^−11^*. For the purpose of deciding if the testing sample is positive, we try to compare it with the other lncRNA–miRNA pairs in the dataset whose interactions are un-confirmed. To do so, we sorted these pair samples and determined the rank of the testing sample among all the 209 152 unidentified samples. If it obtains a higher rank than a given threshold, the testing sample would be considered positive.

For each different threshold set in the experiments, we obtained corresponding true positive rates (TPRs, sensitivity) and FPRs (1−specificity) where the sensitivity and specificity denote the percentage of testing samples with respectively higher and lower ranks than the given thresholds. In addition, we also obtained the ROCs (receiver operating curves) by plotting TPR versus FPR at different thresholds and computed the values of the AUCs. The AUC values lie between 0.5 and 1 where 0.5 denotes a purely random prediction and 1 denotes a perfect performance. The best prediction model built by EPLMI achieved a reliable prediction performance with AUC of 0.8522.

Using the *5-fold cross validation*, all known lncRNA–miRNA interaction data were randomly divided into five subsets of roughly the same size and in each of a series of experiments, four would be used as training samples and the remaining data subset was used as testing samples. As was the case with LOOCV, we obtained the ROC curve for each round of 5-fold cross validation and computed the average value of the AUC. To avoid any bias caused by random partitioning of data subsets, we repeated the random sampling of data 50 times. As a result, we found that the best prediction model obtained by EPLMI achieved an average AUC of 0.8447 ± 0.0017.

Hence, both LOOCV and 5-fold cross validation demonstrate the reliable performance of EPLMI. In Supplementary Table S1, we provide the lists of candidate lncRNAs as predicted by EPLMI for each kind of miRNA. We anticipate that those candidates with higher ranks would be confirmed by the experimental observation in the future.

Considering that different target lncRNAs of each single miRNA have a competitive relationship and that there is still little effort made to analyze such competiveness for sequestering miRNAs, we also released the predicted scores of the known lncRNA–miRNA interactions in Supplementary Table S2. We assume that those lncRNA–miRNA pairs that share a tight relationship in their regulation network tend to be more stable and are, therefore, more competitive in nature.

### 4.3 Comparison among different kinds of RNA-similarity

Apart from the expression profiles, there are other kinds of information, such as target genes of miRNAs, putative biological functions and nucleotide sequence data, which help to describe the features of lncRNA and miRNA. In this section, we further explore two types of such information which are related to RNA-similarity: (i) RNA functional similarity and (ii) RNA sequence similarity. With EPLMI, they can be used to predict lncRNA-miRNA interactions.

**Fig. 4.**
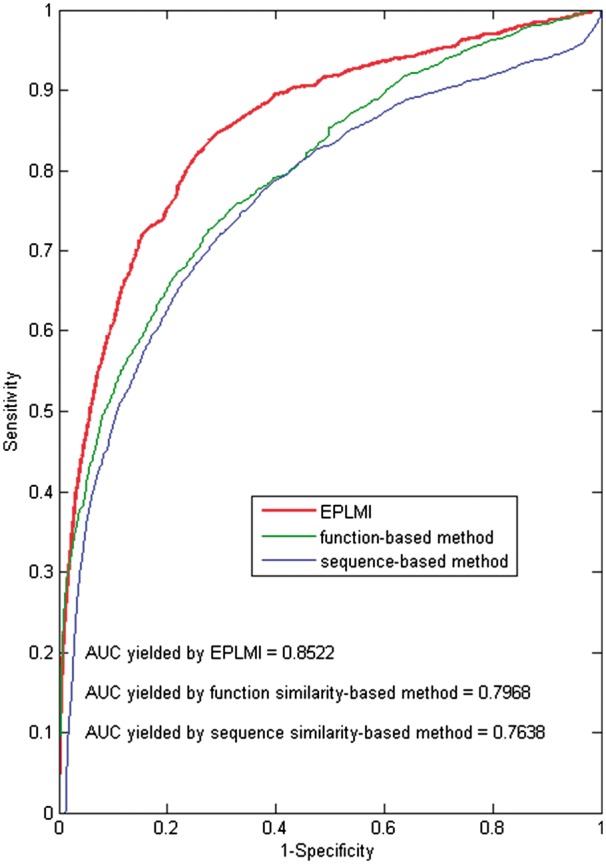
Performance comparison among three kinds of RNA similarity, i.e. expression profile-based, biological function-based and sequence-based similarities, by using the method of EPLMI

To evaluate their usefulness for such purpose, LOOCV and 5-fold cross validation were implemented in this comparison experiments and the results are analyzed and discussed here (see Fig. 4 and Table 1).

**Table 1.** Performance comparison among three kinds of RNA similarity by using EPLMI in the framework of 5-fold cross validation

Expression profile-based similarity	Biological function-based similarity	RNA sequence-based similarity
0.8447 ± 0.0017	0.7608 ± 0.0011	0.7890 ± 0.0014

With regard to (i), recent efforts have been made to predict the biofunctional roles of ncRNAs but the results remain to be categorically proven. To avoid any bias in the prediction of miRNA functions, we used the data of miRNA–target gene associations to measure how functionally similar two miRNAs are. As with the functional similarity of lncRNAs, we simply followed the function annotations of lncRNA based on predictions made by previous work (Zhao *et al.*, 2016).

From the results, the prediction models built by EPLMI using RNA functional similarity and RNA sequence similarity yielded, respectively, AUCs of 0.7968 and 0.7638 in LOOCV experiments. For the *5-fold cross validation* experiments, we obtained average AUCs of 0.7608 ± 0.0011 and 0.7890 ± 0.0014 by using RNA functional similarity and sequence similarity, respectively.

The results of both leave-one-out and 5-fold cross validation demonstrate that the uses of functional similarity and sequence similarity of RNAs are less effective than the use of similarity based on expression profiles. The reason may lie in the fact that the biological roles played by lncRNAs can be so diverse and many lncRNAs may not have appreciable functions which can be described by the known annotations. Hence, the putative lncRNA functional similarity based on coding–non-coding co-expression network may not be accurate and comprehensive enough for this measurement. Furthermore, the size of lncRNA sequences can be very different and the length of the lncRNAs used in this work range from 73 to 59 462. For this reason, simply implementing pairwise sequence global alignment by using a dynamic programming algorithm may not be effective in the measuring of how biologically similar two lncRNAs are or how similar the regulation patterns of two lncRNAs would be. This is because miRNAs are usually sequestered by small-binding sites in lncRNA.

Besides, there are increasing evidence that the expression of lncRNAs is tightly regulated and their expression profiles are important markers for the developmental stage and the disease state. Considering this noteworthy feature of lncRNA, the information of lncRNA expression profiles is considered useful for effectively depicting the correlation of lncRNAs in their miRNA-mediated regulation patterns.

### 4.4 Comparison with different prediction methods

To further evaluate the performance of EPLMI, we compared it with some classical prediction methods by using the same expression profile-based similarity. As the models built by EPLMI uses a network-based method through two-way diffusion, we here explore another kind of network-based method, the Katz measure, which is initially proposed for link prediction problem in social network and extensively used in a diversity of bioinformatics problems.

Further, as the prediction task in this work can be solved as a matrix-completion problem, two main kinds of recommendation algorithms were further investigated. Specifically, two kinds of memory-based collaborative filtering (i.e. lncRNA-based CF and miRNA-based CF) and two kinds of model-based methods [i.e. singular value decomposition (SVD) and latent factor model (LFM)] were implemented for the prediction of lncRNA–miRNA interactions.

**Fig. 5.**
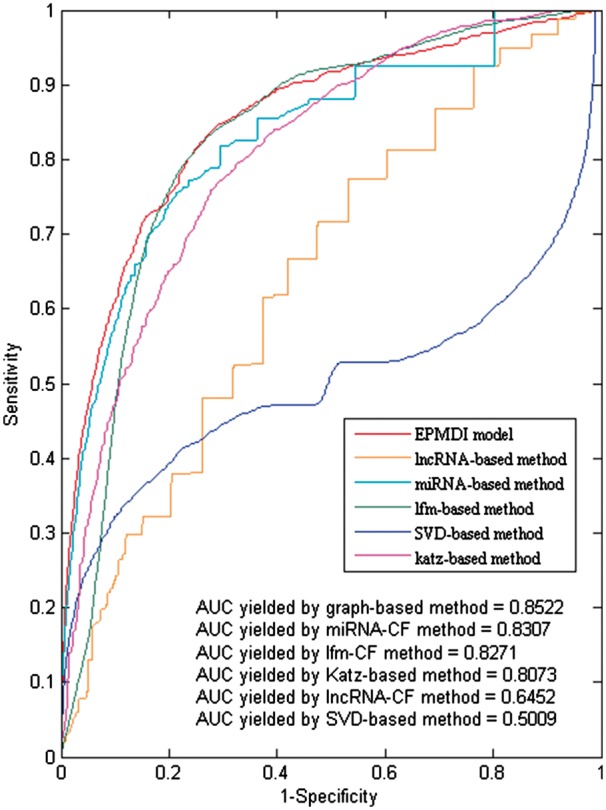
Performance comparison of EPLMI with five different kinds of classical methods by using the same RNA expression profile-based similarity

From the experimental results, it is noted that EPLMI model yielded the best performance among six different algorithms we adopted for comparison using the LOOCV and 5-fold cross validation methods. Specifically, lncRNA-based CF, miRNA-based CF, SVD, LFM and Katz method respectively yielded AUCs of 0.6452, 0.8307, 0.5009, 0.8271 and 0.8073 in LOOCV, and average AUCs of 0.6359 ± 0.0024, 0.8235 ± 0.0015, 0.4967 ± 0.0340, 0.8253 ± 0.0024 and 0.7439 ± 0.0017 in 5-fold cross validation (see Fig. 5 and Table 2). When compared with the other algorithms, the outstanding performance of EPLMI demonstrates that it has reliable prediction performance for large-scale lncRNA–miRNA interactions by well incorporating the information resources of expression profiles.

**Table 2.** Performance comparison among different methods by using RNA expression profile-based similarity in the framework of 5-fold cross validation

Method	5-fold cross validation
lncRNA-based CF	0.6359 ± 0.0024
miRNA-based CF	0.8235 ± 0.0015
SVD-based method	0.4967 ± 0.0340
Katz-based method	0.7439 ± 0.0017
Basic LFM	0.82 53 ± 0.0024
EPLMI	0.8447 ± 0.0017

## 5 Discussion and conclusion

Even though lncRNA–miRNA interactions is becoming known to be very important for dissecting various bio-mechanisms, current knowledge and data on lncRNA–miRNA interaction that have been identified is still limited. Apart from a few sequence-based miRNA target prediction tools that mainly follow the prediction of target genes/mRNA, little effort has been made to predict lncRNA–miRNA interactions on a large scale. Based on accumulating experimental observations, the close relationship between the interaction patterns of ceRNAs and their relative expression levels has been highlighted. In this work, motivated by recent advances in the synergistic actions of lncRNAs, we analyzed statistically the patterns of large scale lncRNA–miRNA interaction network in the perspective of expression profiles. Consequently, we discovered that lncRNAs/miRNAs interacting with the same single miRNAs/lncRNA tend to have similar expression profiles. Based on this finding, we propose the first computational technique, EPLMI, to build models for predicting large-scale lncRNA–miRNA interaction network based on a novel graph-based diffusion algorithm. The basic assumption made by EPLMI is that lncRNAs with similar expression profiles tend to collaboratively interact with miRNAs with similar expression profiles, and vice versa. By using the latest dataset of lncRNA–miRNA interactions, the experimental results obtained with EPLMI, along with a series of comparison results, demonstrate that it can be a very reliable method.

We believe that EPLMI can yield important insights into future research on ceRNA regulation networks. Unlike traditional prediction tools for miRNA–mRNA interactions, EPLMI do not focus on binding sites of miRNA in target RNAs considering that the number of binding rules of MREs are still very limited due to naturally imperfect pairing and that purely computing free-energy could yield a high rate of false positives. Instead, EPLMI predicts lncRNA–miRNA interactions by making use of the collaborative effects of both lncRNAs and miRNAs and the similarities of lncRNAs and miRNAs. By using the expression profiles of lncRNAs and miRNAs, EPLMI can yield the interaction possibility for each lncRNA–miRNA pair in one-shot and it can, therefore, have a wide-range of applications.

In addition to the above, EPLMI can offer preliminary knowledge for two other prediction problems that we propose for future work. The first one is to predict the indirect lncRNA–lncRNA interactions. It is reported that indirect interactions occur frequently in ceRNA network where two ceRNAs can crosstalk via a third transcript. As EPLMI focuses on the common pattern of lncRNAs interacting with the same single miRNAs, the lncRNAs predicted to interact the same miRNAs with high scores may tend to have an indirect interaction.

The second one is to measure how competitive the lncRNAs are to sequester a specific kind of miRNA. Target lncRNAs may coexist as competing ceRNAs and the effectiveness and number of their MREs are not always equal, leading to different competitive status. By implementing EPLMI, those links of known lncRNA–miRNA interactions that have bigger weights could be considered as more common and biologically important than the others and therefore the lncRNAs in these interactions may have higher priority to interact with the miRNAs in order to remain biologically stable. In other words, for the known lncRNA–miRNA interactions, the lncRNAs obtaining higher scores predicted by EPLMI may be more competitive in their interaction with miRNAs.

Despite the effectiveness of EPLMI as discussed above, it should be noted that EPLMI has some limitations. As EPLMI makes prediction mainly based on datasets with known lncRNA–miRNA interaction, it may suffer from possible prediction-bias caused by imbalanced learning samples. lncRNA/miRNA that are well-studied tend to obtain a higher prediction scores since they have more links in known lncRNA–miRNA interaction network. In addition, it should also be noted that EPLMI is not applicable to new types of lncRNA/miRNA that are without expression profiles as they do not have any links in known lncRNA–miRNA interaction network.

## Funding

Yu-An Huang was supported by the National Natural Science Foundation of China under Grant No. 61702424. Zhu-Hong You was supported by the National Natural Science Foundation of China under Grant No. 61572506.

*Conflict of Interest*: none declared.

## Supplementary Material

Supplementary Data
